# Hubble parameter estimation via dark sirens with the LISA-Taiji network

**DOI:** 10.1093/nsr/nwab054

**Published:** 2021-04-01

**Authors:** Renjie Wang, Wen-Hong Ruan, Qing Yang, Zong-Kuan Guo, Rong-Gen Cai, Bin Hu

**Affiliations:** Department of Astronomy, Beijing Normal University, Beijing 100875, China; CAS Key Laboratory of Theoretical Physics, Institute of Theoretical Physics, Chinese Academy of Sciences, Beijing 100190, China; School of Physical Sciences, University of Chinese Academy of Sciences, Beijing 100049, China; College of Engineering Physics, Shenzhen Technology University, Shenzhen 518118, China; CAS Key Laboratory of Theoretical Physics, Institute of Theoretical Physics, Chinese Academy of Sciences, Beijing 100190, China; School of Physical Sciences, University of Chinese Academy of Sciences, Beijing 100049, China; School of Fundamental Physics and Mathematical Sciences, Hangzhou Institute for Advanced Study, University of Chinese Academy of Sciences, Hangzhou 310024, China; CAS Key Laboratory of Theoretical Physics, Institute of Theoretical Physics, Chinese Academy of Sciences, Beijing 100190, China; School of Physical Sciences, University of Chinese Academy of Sciences, Beijing 100049, China; School of Fundamental Physics and Mathematical Sciences, Hangzhou Institute for Advanced Study, University of Chinese Academy of Sciences, Hangzhou 310024, China; Department of Astronomy, Beijing Normal University, Beijing 100875, China

**Keywords:** gravitational waves, Hubble parameter, super massive black hole

## Abstract

The Hubble parameter is one of the central parameters in modern cosmology, and describes the present expansion rate of the universe. The values of the parameter inferred from late-time observations are systematically higher than those inferred from early-time measurements by about $10\%$. To reach a robust conclusion, independent probes with accuracy at percent levels are crucial. Gravitational waves from compact binary coalescence events can be formulated into the standard siren approach to provide an independent Hubble parameter measurement. The future space-borne gravitational wave observatory network, such as the LISA-Taiji network, will be able to measure the gravitational wave signals in the millihertz bands with unprecedented accuracy. By including several statistical and instrumental noises, we show that, within a five-year operation time, the LISA-Taiji network is able to constrain the Hubble parameter within $1\%$ accuracy, and possibly beats the scatters down to $0.5\%$ or even better.

## Teaser

The future space-borne gravitational wave observatory network will be able to reveal the puzzle of the present cosmic expansion rate with unprecedented accuracy.

## INTRODUCTION

The measurement of the Hubble parameter has reached a crossroad [1]. The values obtained from early-time observables such as the cosmic microwave background (CMB) [2] or the big bang nucleosynthesis plus baryon acoustic oscillation [3] are indirect, because to get *H*_0_ from those measurements one has to assume a cosmological model. Although these measurements are more precise compared with the late-time distance ladder [4,5], in this way the resulting *H*_0_ is cosmological model dependent. The distance ladder is a direct *H*_0_ measurement. However, generally, it has more serious systematics, such as the reddening of the cepheid or red-giant branch stars, metallicity effects, etc. [4,5]. Hence, the resulting values might be miscalibrated due to the aforementioned astro-physical issues. A new independent *H*_0_ measurement whose accuracy is better than $2\%$ is crucial in order to judge the current discrepancy [6,7]. Once this $2\%$ precision level is achieved, we give priority to understanding the systematics, especially the unknown ones, rather than simply to increasing the sample volume.

With self-calibration by the theory of general relativity, gravitational waves (GWs) from compact binary coalescence (CBC) events open a completely novel observational window for *H*_0_ determination [8–12]. Depending on whether or not the events are associated with electromagnetic (EM) counterparts, GW events can be categorized into bright sirens [13,14] and dark sirens [15–17]. The former demand fairly good synergies, which are extremely challenging for high redshift CBC events; while the latter, which do not rely on transient measurements, ask for a precise sky localization to reduce the number of possible host galaxies. Since the GW siren is a completely independent measurement, its result will suffer from different systematics. Hence, it can shed some light on the Hubble tension. Resolving this tension will lead to important implications. If the result from the GW siren is consistent with that from the early-time measurements, such as the CMB, it would imply that the current understanding of distance ladder systematics is not enough and that the concordance model ΛCDM still works. On the other hand, if the result from the GW siren agrees with that from the distance ladders, one needs to revise the ΛCDM model and there must exist some new physics beyond the standard model of cosmology. This is because several CMB experiments (including both space mission and ground-based telescopes), such as Planck [2], SPT [18] and ACT [19], are consistent with each other. Each of these experiments has special designs in itself. Hence, they have different systematics.

The Laser Interferometer Space Antenna (LISA) [20], a space-borne gravitational wave observatory, consists of three spacecrafts in an equilateral triangle configuration. The separation distance between the spacecrafts is about 2.5 million kilometres. The LISA constellation is in a heliocentric orbit behind the Earth by about 20°. Taiji [21] is a gravitational wave space facility proposed by the Chinese Academy of Sciences, with a separation distance of 3 million kilometres in a heliocentric orbit ahead of the Earth by about 20°. The LISA-Taiji network [20,21] will be able to localize the CBC events with unprecedented accuracy [22]. As demonstrated previously, this advantage could help improve the Hubble constant determination.

In this article we forecast the ability of estimating the Hubble parameter by using GW siren data from the future space-based GW observatories. Unlike stellar-mass binary black holes detected with LIGO/Virgo [23], for which the merger rate is observationally measured, there is no conclusive observational evidence for merging massive binary black holes (MBHs). The models [24,25] adopted in this article provide viable theoretical predictions to our knowledge, and are also extensively studied in the literature. The models are built by combining the cosmological galaxy formation history with the massive black hole binary (MBHB) formation dynamics. Specifically, the models follow the evolution of baryonic structures along a dark-matter merger tree according to the extended Press-Schechter formalism that is calibrated by *N*-body simulations. Besides MBHs, the baryonic ingredients of the model include the hot unprocessed inter-galactic medium, the cold metal-enriched inter-stellar medium, the stellar galactic disk, the stellar spheroid, the nuclear gas and the nuclear star cluster. In the next section, we highlight two of the most relevant aspects with GW emissions, namely, black hole seedings and time delays.

## MODELS

We consider three different massive black hole formation models with different black hole seedings and time delays. The ‘light-seed’ scenario assumes that the black hole seeds are the remnants of population III stars (PopIII) with typical initial masses centered at 300*M*_⊙_, which is called the ‘PopIII’ model. In the ‘heavy-seed’ scenario (assuming a critical Toomre parameter of *Q*_*c*_ = 3), MBHs arise from the collapse of protogalactic disks and already have masses around 10^5^*M*$_{\odot}$ at high redshifts $z$ = 15–20. Depending on whether or not delays exist between MBHs and galaxy mergers, these ‘heavy-seed’ models are referred to as ‘Q3d’ and ‘Q3nod’, respectively.

In ‘PopIII’ and ‘Q3d’ models, after the dynamical friction phase, several hardening mechanisms are included. In gas-rich environments, the nuclear gas viscosity drags the merger of MBHB behind the merger galaxies. The typical delay is about 10 ∼ 100 Myr. In gas-poor environments, three-body interactions with stars dominate the hardening process. This brings MBHs together on a time scale of about 5 Gyr. If a MBHB stalls at about a persec separation, a MBH triple system may be formed when a succeeding galaxy merger occurs. The typical delay is about 100 Myr. This mechanism seems to work effectively only for heavy systems with masses >10^6^–10^7^*M*$_{\odot}$; otherwise, the lightest MBH may also be ejected via the gravitational slingshot mechanism before the triple interactions trigger the merger of the inner binary. The details of the time delay prescriptions can be found in [26]. One can view ‘Q3d’ and ‘Q3nod’ as the conservative and optimistic limits of the ‘heavy-seed’ scenario.

For each of the three models, we consider two types of mission configuration (‘the LISA-Taiji network’, ‘Taiji only’) and three different observation times (one year, three years, five years). For each combination of the model, mission configuration and observation time, we generate 40 sets of simulations, including both the instrumental noise [27,28] and lensing noise [29,30]. Each set of simulations contains a few tens or a few hundreds of CBC events according to different MBH formation models. For each simulated CBC event, we estimate the posterior probability of the luminosity distance from the frequency-domain GW strains by using the Fisher information matrix method, which will be briefly mentioned in the following section.

In order to determine *H*_0_, we also need the redshift information from the host galaxy. To this end, we sample galaxies uniformly in the co-moving volume with a number density of 0.02 Mpc^−3^, according to the model [24]. The adopted values of the galaxy number density are located in the middle of the observational error bars (see Fig. 1 of [24]). We verified that, within the observational uncertainty range (2 × 10^−3^, 6 × 10^−2^), except for the blue events, the *H*_0_ estimations from all other event types (see the definition of the different types of events in the subsequent context) are insensitive to the number density choice, due to the excellent sky localization. Then, we locate the possible host galaxies within $99\%$ ellipsoidal contours in the three-dimensional parameter space spanned by the luminosity distance and observation solid angles. For each of the host galaxy candidates, we assume that their redshift uncertainties are negligible. Finally, we present the Hubble parameter estimations based on these 720 sets of simulations. The flat ΛCDM model with *H*_0_ = 67.74 and Ω_*M*_ = 0.3 is taken as our fiducial cosmological model. The following results will not significantly rely on the fiducial cosmological model, especially for the local CBC events. It might be worth noting here that one should pay attention to the accuracy of the Hubble parameter *H*_0_ through our simulations, rather than the resulting *H*_0_ value itself in this work.

**Figure 1.**
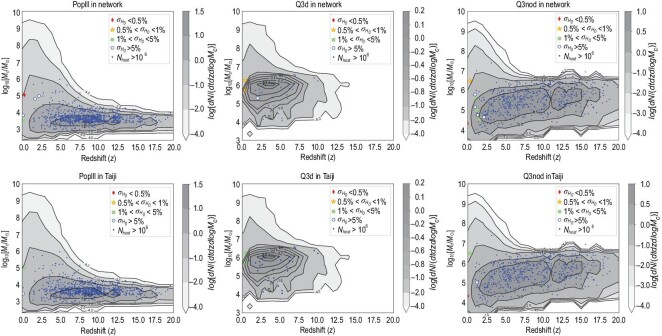
The simulated merger event rate distribution of MBHBs in redshift and chirp mass within the five-year observation time of the LISA-Taiji network and Taiji-only mission. Red diamonds ($\sigma _{H_0}/H_0<0.5\%$), yellow stars ($0.5\%$–$1\%$), green squares ($1\%$–$5\%$) as well as open blue circles ($>\!5\%$) are the classified dark sirens according to their Hubble parameter estimation accuracies. The filled blue circles are the unqualified dark sirens whose possible host galaxy numbers are more than 10^6^ due to the poor sky localization. The background gray contours are the theoretical MBHB merger event rate distributions. The first row displays the results of the LISA-Taiji network, while the second row displays those for the Taiji-only case. The first, second and third columns display the predictions from three different MBH models, namely PopIII, Q3d and Q3nod, respectively.

## RESULTS

**Figure 2.**
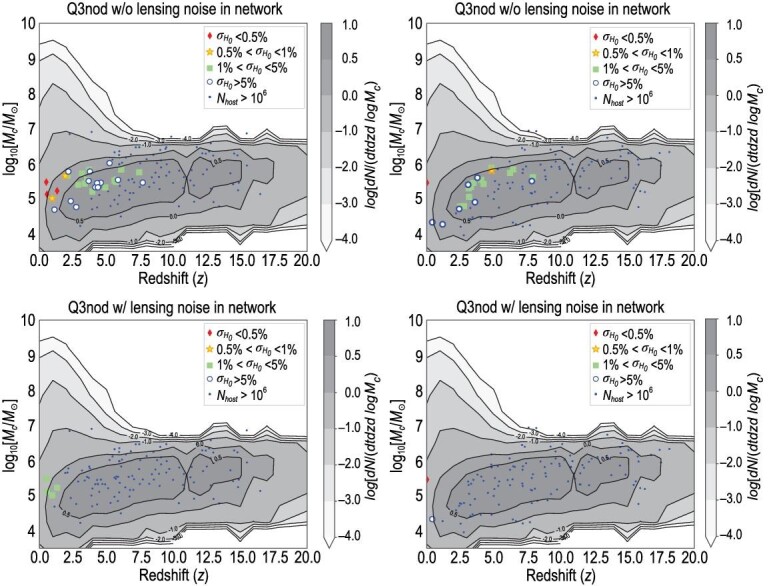
Event distribution of dark sirens after a one-year observation of the LISA-Taiji network with and without lensing noise. We show four mocked MBHB catalogs with one-year LISA-Taiji network observations. The left and right columns display two individual realizations of Q3nod+network. The top-left panel has a few diamond events with $z$ > 0.5; while in the top-right panel there is only one diamond at $z$ = 0.02. The first row displays the mocks without lensing noise. The second row displays the mocks with lensing noise.

In Fig. 1, we show one typical set of five-year simulations in the LISA-Taiji network (the first row) as well as the Taiji-only mission (the second row). By the time of Taiji/LISA data collection, several *H*_0_ measurements will hopefully achieve $1\%$ precision [6,31]. Hence, we classify all the qualified dark siren events into four groups, namely diamond, gold, green and blue. They correspond to Hubble parameters with $<\!\!0.5\%$, $0.5\%$–$1\%$, $1\%$–$5\%$ and $>\!5\%$ accuracies, respectively. Firstly, one can see that in all six panels the number of qualified events is less than ten. This is because the nominal *H*_0_ accuracies are extremely challenging. Only events whose luminosity distance uncertainties are below percent levels can qualify. Secondly, all the qualified events are distributed below redshift $z$ = 2.5. This is due to the lensing noise that will be demonstrated later. Thirdly, the LISA-Taiji network can improve the results significantly, compared to the Taiji-only case. The upper and lower panels of each column display results from the same CBC realizations. Their differences lie in the mission configuration. Taking the Q3d column as an example, the Taiji-only mission can capture two green events after a five-year observation. In addition to capturing another blue event at a redshift of 2, the LISA-Taiji network is able to upgrade the two green events in the Taiji-only mission to gold. Last but not least, all diamond events are distributed in the very local universe. This is also because, as long as $z$ > 0.35, the distance uncertainties induced by the unavoidable gravitational lensing do not meet the *H*_0_ accuracy request. In order to explain this more clearly, we show the event distribution in the cases with and without lensing noise in Fig. 2. The two panels in the first row display the event distribution without lensing noise, with two individual realizations of Q3nod+network shown in the left and right panels. The top-left panel has a few diamond and gold events in the redshift range $z$ > 0.5, while in the top-right panel there is one diamond event at $z$ = 0.02 and one gold event in the high redshift ($z$ = 4.86). One can see that, without considering lensing noise, the LISA-Taiji network could detect the qualified events all the way up to $z$ ≃ 8. The two panels in the second row display the event distribution with lensing noise. Comparing with the top-left panel, in the bottom-left panel all the original green and blue events fail the qualifications. Only the original three diamond events and one gold event survive, downgraded to green. However, the diamond in the top-right panel still keeps its identity in the bottom-right panel because lensing noise is negligible in the nearby universe.

**Figure 3.**
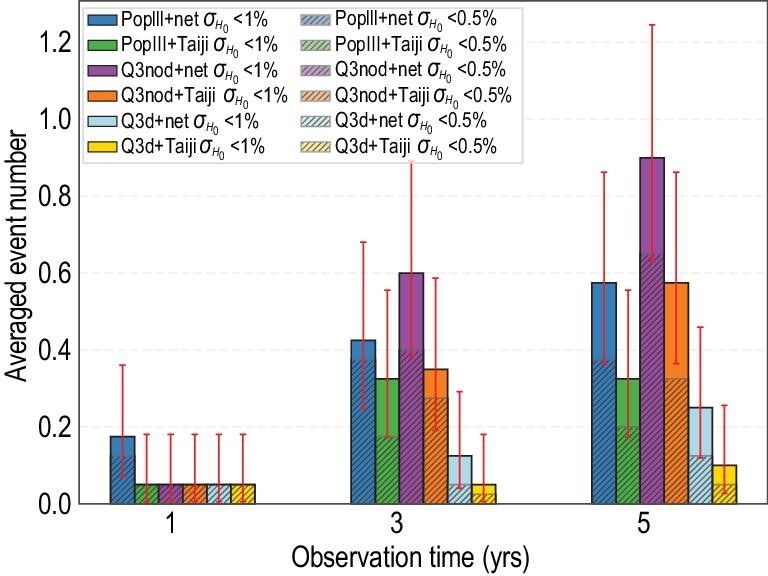
Averaged event number over one-year, three-year and five-year observation times. Blue, green, purple, orange, cyan and yellow histograms denote the averaged event number in PopIII+network, PopIII+Taiji, Q3nod+network, Q3nod+Taiji, Q3d+network and Q3d+Taiji, respectively. The unshaded histograms denote the dark sirens with *H*_0_ accuracies better than $1\%$, namely diamond+gold events. The red error bars denote the $95\%$ confidence interval by assuming a Poisson distribution. These error bars merely account for the statistical errors. The shaded histograms denote the dark sirens with *H*_0_ accuracies better than $0.5\%$, namely diamond only.

In Fig. 3, we show the averaged event numbers for the PopIII, Q3nod as well as Q3d models over one-year, three-year and five-year observation times, respectively. In order to suppress the statistical errors, we compute each of the average numbers over 40 sets of simulations. From the statistics of the one-year and three-year observations, we cannot guarantee capturing one diamond or gold event with $95\%$ confidence level. After the five-year network observation, for the Q3nod model, the averaged event number with *H*_0_ accuracy better than $1\%$ could reach 0.9 and its $95\%$ confidence interval will up-cross unity. We will very probably capture one gold or diamond event after a five-year network observation. Comparing the shaded histogram (only diamond) with the unshaded histogram (diamond+gold) of Fig. 3, we can see that the possibility of capturing a diamond event is actually higher than that of a gold event. Again, this is still because of lensing noise. For the PopIII model, the averaged event number accumulated in the network after five years is about 0.58, with the $95\%$ confidence interval in 0.36–0.86. For the Q3d model, the averaged event number after five-year monitoring by the network is $0.25^{+0.20}_{-0.13}$. The corresponding five-year numbers in the Taiji-only mission for both the PopIII and Q3nod models are about two-thirds of those in the LISA-Taiji network. The Q3d events number in a five-year Taiji-only mission is about half of the LISA-Taiji network case. This implies that, with the Taiji-only mission, we lack confidence in capturing at least one diamond or gold event during the five-year observation. For the green ($1\%<\sigma _{H_0}<5\%$) events, the averaged numbers after a five-year network observation are 4.05, 0.88, 0.38 in the Q3nod, PopIII and Q3d models, respectively. For the blue ($\sigma _{H_0}>5\%$) events accumulated in the five-year network observation, the numbers are 8.00, 3.60, 1.23. The corresponding numbers of green (blue) events for the five-year Taiji-only mission are 0.58, 0.23, 0.25 (1.73, 0.23, 0.20), respectively, for the three models. For elaborated statistics, we refer the reader to Table 3 of the online supplementary material.

**Figure 4.**
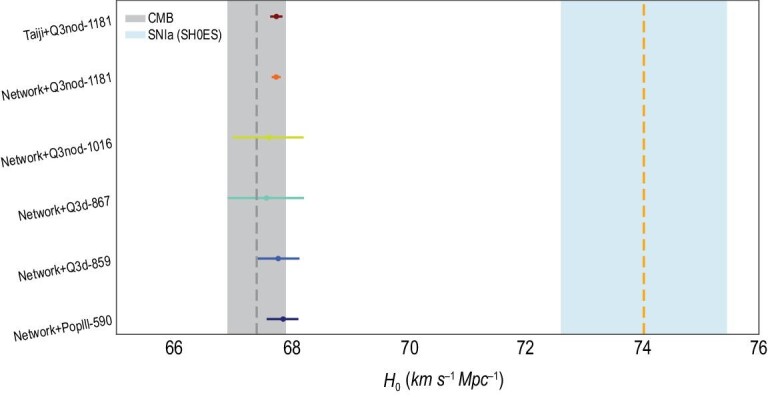
Error estimation of the Hubble parameter from the diamond and gold events in the LISA-Taiji network and Taiji-only mission after a five-year observation time. The vertical gray (*H*_0_ = 67.4 ± 0.5 km s^−1^ Mpc^−1^) and cyan (*H*_0_ = 74.03 ± 1.42 km s^−1^ Mpc^−1^) bands denote the present *H*_0_ results from the cosmic microwave background (Planck [2]) and SNIa (SH0ES [4]), respectively. The fiducial value of the Hubble parameter is *H*_0_ = 67.74. The vertical axes are labeled as ‘mission+event ID’. Among these six events, Net+Q3nod-1181, Taiji+Q3nod-1181 and Net+PopIII-590 are the diamond events. The rest are gold events.

In Fig. 4, we show the detailed *H*_0_ results from diamond and gold events within the five-year observation, which have already been shown in Fig. 1. The event Q3nod-1181 can qualify as the diamond in both the LISA-Taiji network and Taiji-only mission. The former gives $H_0=67.73^{+0.08}_{-0.08}$, while the latter gives $H_0=67.74^{+0.10}_{-0.10}$. They are both $0.1\%$ measurements. This is because Q3nod-1181 is located only at $z$ = 0.01. Both the LISA-Taiji network and Taiji-only mission are able to detect it with an extremely high signal-to-noise ratio (SNR ∼ 10^5^). To ensure that such a local event is not a statistical fluke, we checked the redshift distribution of diamond events over 40 sets of simulations under the ‘Q3nod+network+5yrs’ configuration. We found that there are 5 out of 26 diamond events whose redshift equals 0.01. Besides, there are another three diamond events whose redshifts are below 0.03. Such local diamond events are typical in the ‘Q3nod’ model. As for the PopIII-590 event, the LISA-Taiji network can detect it as a diamond event ($H_0=67.85^{+0.26}_{-0.28}$, $0.4\%$ accuracy) with SNR ∼895. However, the Taiji mission can merely detect it as a green event ($H_0=67.81^{+1.08}_{-1.02}$, $1.6\%$ accuracy) with much lower SNR (∼564). Moreover, we can also tell the difference between the two mission configurations by the sky area and the number of possible host galaxies. For PopIII-590, these two numbers in the LISA-Taiji network are 0.004 deg^2^ and 3 galaxies, while in the Taiji-only mission, they are 0.5 deg^2^ and 1022 galaxies. From this example, we can clearly see that the network cannot only double the SNR, but also significantly improve the sky localization (reduce the number of possible host galaxies). These two aspects could help the measurement of the Hubble parameter by using dark sirens. Besides the diamond events, there are another three gold events, namely Q3nod-1016, Q3d-867 as well as Q3d-859, which could only be observed by the LISA-Taiji network. Furthermore, there are three green and four blue events in the five-year network observation (see the top-right panel of Fig. 1). For detailed statistics, we refer the reader to Tables S1 and S2 of the online supplementary material. Although the green and blue events are not our major concerns, by combining these classified events, we can further reduce the *H*_0_ error bars by at least $20\%$ ($H_0=66.61^{+1.80}_{-2.28}$ for joint green, $H_0=65.41^{+2.70}_{-3.60}$ for joint blue) with respect to the best individual cases in each category ($H_0=67.08^{+2.28}_{-2.46}$ for the best green, $H_0=66.31^{+3.42}_{-4.05}$ for the best blue). These can be seen in Fig. S2 of the online supplementary material.

## DISCUSSION

The GW siren is an independent *H*_0_ measurement procedure. Through the GW waveform, one is able to determine the luminosity distances to the GW sources. Once the redshifts of GW sources are known through the bright sirens or dark sirens, one can obtain a relation between the distance and redshift, through which *H*_0_ is inferred. This *does not* mean that all the inferred *H*_0_ values are cosmological model (e.g. ΛCDM) independent. In principle, if the Friedmann equation is used in the *H*_0_ inference, the method is cosmological model dependent; otherwise, it is not. One example of the model-independent method is the SNIa distance ladder, in which the Hubble function or luminosity distance is Taylor expanded in terms of the redshift. As shown in [32], the maximum redshift of this approach can be extended to $z$_max_ = 0.4. A similar method can be applied to the GW sirens. In Table S1 of the online supplementary material, we list all the qualified dark sirens in Taiji. One can see that six out of seven events are distributed below redshift 0.4. Moreover, in Table S2 of the online supplementary material, all of the diamond and gold events in the LISA-Taiji network are distributed below redshift 0.4. These local events can be used to infer the *H*_0_ value via a cosmological model-independent method. However, there are some blue and green events from redshifts close to or higher than 1. To utilize these data to infer the *H*_0_ value, one has to assume a background cosmological model. However, due to the poor quality of these data points, the resulting *H*_0_ estimation from these events is a marginal result.

GW cosmology, as a new exciting field, has a lot of unknowns in both theoretical modelings and observational systematics. The results presented above are based on a simplified model setup. There are lots of informative phenomena that we decide to turn a blind eye to. First, we assume that all MBHB mergers are dark. As shown in our studies, the most important MBHB mergers for measuring *H*_0_ are indeed those in the nearby universe. For them, the EM counterpart observation may be possible [33]. If EM counterparts can be identified, it will help to improve the sky localization significantly. Second, we do not consider the galaxy clustering effect. The uniform distribution will hold on average over sufficiently large volumes. However, in the small localization ellipsoid, the clustering could help to reduce the *H*_0_ error bars [13,15,17,34,35]. The clustering makes the redshift distribution more concentrated. Since the final *H*_0_ posterior is the sum over all possible redshifts, the narrower the redshifts are distributed, the faster the posterior will converge. In addition, although the sirens (both the bright and dark) method requires the redshift information, it does not require uniquely identifying the host galaxy, because the redshift is a smoothly varying quantity. Large-scale structure predicts that fainter galaxies follow the clustering pattern of the more luminous galaxies. Hence, if the MBHB localization ellipsoid is small enough, we may uniquely identify the central bright galaxy of the cluster where the true host faint galaxies reside. In this case, we are actually able to upgrade the dark sirens to bright sirens. Third, in order to avoid any theoretical bias, we do not utilize any other galaxy properties besides the redshift. This is because our current understanding of the relationship between MBHs and dwarf galaxies is still uncertain. If we could improve our knowledge on these aspects, we can aim at a particular type of galaxy instead of all galaxies in the three-dimensional contours. As for the redshift uncertainties and the galaxy incompleteness, we have means to mitigate these problems. Unlike stellar binary black holes, there are fewer MBHB populations. With the help of the space-based GW observatory network, we are able to localize each of them in a small area, such as <10 arcmin^2^. Instead of using pre-existing galaxy catalogs, we could conduct deep optical and radio EM follow-ups for the limited diamond and gold events. For (dwarf) galaxies with stellar masses 10^8^*M*$_{\odot}$ (corresponding to the central MBH with masses 10^5^*M*$_{\odot}$) at a luminosity distance of 1500 Mpc, the *K*-band luminosity is about 24 magnitude, which is completely visible for an up-coming spectrograph observation, such as the Thirty Meter Telescope [36]. Based on these arguments, we believe we present an almost risk-free science case for the future space-borne GW mission.

## METHODS

In this section, we present some essential aspects in the methodology of estimating *H*_0_.

### Fisher matrix

In order to simplify the calculation, we adopt the restricted post-Newtonian (PN) approximation of the GW waveform for the non-spinning MBHB [37]. For a non-spinning MBHB at a luminosity distance *d*_*L*_, with component masses *m*_1_ and *m*_2_, total mass *M* = *m*_1_ + *m*_2_, symmetric mass ratio η = *m*_1_*m*_2_/*M*^2^ and chirp mass *M*_*c*_ = η^3/5^*M*, the frequency-domain version of the strain is given by [22,38],

(1) \begin{eqnarray*} \tilde{h}(f)&=&-\bigg (\frac{5\pi }{24}\bigg )^{1/2}\bigg (\frac{GM_c}{c^3}\bigg )\bigg (\frac{GM_c}{c^2 D_{{\rm eff}}}\bigg )\nonumber\\ &&\times\,\,\bigg (\frac{GM_c}{c^3}\pi f\bigg )^{-7/6}e^{-i\Psi (f\,\,M_c,\eta )},\\ \end{eqnarray*}

where *D*_eff_ is the effective luminosity distance to the source,

(2) \begin{equation*} D_{{\rm eff}}=d_L \bigg [F^{2}_{+}\bigg (\frac{1+{\rm cos}^2 \iota }{2}\bigg )^2+F^{2}_{\times } {\rm cos}^2 \iota \bigg ]^{-1/2} \end{equation*}

with inclination angle ι. The phase Ψ depends on the coalescence time *t*_*c*_ and the coalescence phase φ_*c*_ [39]. In this paper, Ψ is calculated up to the second PN order. The response functions *F*_+_, *F*_×_ depend on the sky direction of source (α, δ) and the polarization angle ψ. For a space-based GW detector such as LISA and Taiji, *F*_+_ and *F*_×_ are functions of frequency [22]. In the calculation, the response functions of LISA and Taiji are obtained from previous work [40] with a stationary phase approximation [41].

The Fisher matrix approach is employed in this paper to determine the uncertainty of parameter measurements for a GW observation. For multiple detectors, the joint Fisher matrix is given by [41,42],

(3) \begin{equation*} \Gamma _{ij}=\bigg (\frac{\partial _i \boldsymbol{d}(f)}{\partial \lambda _i}, \frac{\partial _j \boldsymbol{d}(f)}{\partial \lambda _j}\bigg ), \end{equation*}

where

(4) \begin{equation*} \boldsymbol{d}(f)=\bigg [\frac{^\tilde{h}_1 (f)}{\sqrt{S_1 (f)}\, },\frac{^\tilde{h}_2 (f)}{\sqrt{S_2 (f)}\, },\ldots ,\frac{^\tilde{h}_N (f)}{\sqrt{S_N (f)}\, }\bigg ]^{T} \end{equation*}

and the λ_*i*_ denote the parameters of interest. We consider the nine parameters of a non-spinning MBHB (*M*_*c*_, η, *d*_*L*_, ι, α, δ, *t*_*c*_, φ_*c*_, ψ). Hence, Γ is a nine-dimensional matrix. Here, *S*_*i*_(*f*) is the noise power spectral density (PSD) of the *i*th detector and the $^\tilde{h}_i (f)$ are the frequency-domain GW strains. The noise-weighted inner product in Equation (3) for two functions *a*(*t*) and *b*(*t*) is defined as

(5) \begin{equation*} (a, b)=2\int _{f_{\rm low}}^{f_{\rm up}}\lbrace \tilde{a}(f)\tilde{b}^{*}(f) +\tilde{a}^{*}(f)\tilde{b}(f)\rbrace {d}f.\end{equation*}

The upper cutoff frequency *f*_up_ is chosen as the innermost stable circular orbit (ISCO) frequency *f*_isco_ in the analysis, which is given by

(6) \begin{equation*} f_{\rm isco}=\frac{c^3}{6\sqrt{6}\pi GM}. \end{equation*}

Assuming stationary Gaussian detector noise, the root-mean-square error of λ_*i*_ is given by

(7) \begin{equation*} \sqrt{\langle \Delta \lambda _i^2 \rangle } =\sqrt{(\Gamma ^{-1})_{ii}}. \end{equation*}

In our calculation, we use two Michelson-style data channels and the joint Fisher matrix is a sum of two Fisher matrices.

For a detected source at sky direction (α, δ), the angular resolution is given by [41,42],

(8) \begin{equation*} \Delta \Omega _s=2\pi |{\rm sin}\alpha | \sqrt{\langle \Delta \alpha ^2 \rangle \langle \Delta \delta ^2 \rangle -\langle \Delta \alpha \Delta \delta \rangle ^2}, \end{equation*}

where 〈Δα^2^〉, 〈Δδ^2^〉 and 〈ΔαΔδ〉 are given by the inverse of the Fisher information matrix. The uncertainty of *d*_*L*_ can also be obtained according to Equation (7).

### Lensing noise

The effect of lensing magnification in a GW observation is considered in the analysis. In this paper, we model lensing effect via a stochastic noise in the luminosity distance. The fitting formula of the GW luminosity distance error due to lensing is given by [29],

(9) \begin{equation*} \sigma _{\rm lens}(z)=\frac{\Delta d_L}{d_L}=0.066\bigg [ \frac{1-(1+z)^{-0.25}}{0.25}\bigg ]^{1.8}. \end{equation*}

Hence, Equation (4) can be rewritten as

(10) \begin{eqnarray*} \boldsymbol{d}(f)&=&\Bigg [\frac{^\tilde{h}_1 (f)}{\sqrt{S_1 (f)+S_{1}^{\rm lens}(f)}\, },\nonumber\\ &&\frac{^\tilde{h}_2 (f)}{\sqrt{S_2 (f)+S_{2}^{\rm lens}(f)}\, },\ldots ,\nonumber\\ &&\frac{^\tilde{h}_N (f)}{\sqrt{S_N (f)+S_{N}^{\rm lens}(f)}\, }\Bigg ]^{T} , \end{eqnarray*}

where the PSD of lensing noise for *i*th detector $S_{i}^{\rm lens}(f)$ is given by

(11) \begin{equation*} S_{i}^{\rm lens}(f) = f \cdot \left|^\tilde{}{h}_{i}^{\rm lens} (f) \right|^2, \end{equation*}

and $^\tilde{}{h}_{i}^{\rm lens} (f)$ is obtained from

(12) \begin{eqnarray*} ^\tilde{}{h}_{i}^{\rm lens} (f) &=& \frac{1}{2}\bigg [\frac{^\tilde{}{h}_i(f)}{1-\sigma _{\rm lens}(z)}-\frac{^\tilde{}{h}_i(f)}{1+\sigma _{\rm lens}(z)}\bigg ]\nonumber\\ &\approx &\sigma _{\rm lens}(z)\cdot ^\tilde{}{h}_i(f). \end{eqnarray*}

**Figure 5.**
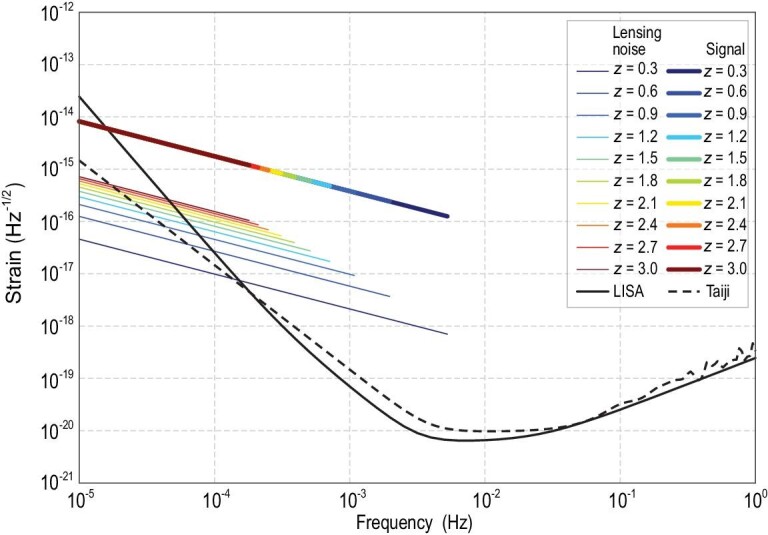
Lensing and instrumental sensitivity curves in LISA and Taiji. The black solid and dashed curves are the instrumental sensitivity curves for LISA and Taiji, respectively. The colored thin curves are the lensing noise of MBHB sources. The colored thick curves are the GW signal strains. Different colors denote different source redshifts.

In Fig. 5, we show the lensing and instrumental sensitivity curves in LISA and Taiji space missions. The black solid and dashed curves are the instrumental sensitivity curves for LISA and Taiji, respectively. The colored thin curves are lensing noise of MBHB sources. The colored thick curves are the GW signal strains. Different colors stand for different source redshifts. One can see that, from the redshift 0.3 to 3, lensing noises dominate over instrumental noises in the frequency range of a few 10^−5^ Hz to a few millihertz. Lensing noise is the major component in the noise budget. Here let us mention that because we want to demonstrate the relative lensing noise amplitude, we normalize all primary GW strain signals from different redshifts, $^\tilde{h}_i(f)$, with the same amplitude. This is why all signal curves align on the same line in Fig. 5.

In this article, we simulate binary coalescence signals $\boldsymbol{d}(f)$ assuming a flat ΛCDM cosmology with *H*_0_ = 67.74 and Ω_*M*_ = 0.3. The sky direction, inclination angle, coalescence phase and polarization angle are randomly chosen in the ranges α ∈ [0, π], δ ∈ [0, 2π], ι ∈ [0, π], φ_*c*_ ∈ [0, 2π] and ψ ∈ [0, 2π]. The coalescence time of these samples is chosen to be *t*_*c*_ = 0, and *f*_low_ in Equation (5) is randomly chosen between 10^−5^ Hz and the ISCO frequency. Moreover, we adopt the noise PSD without foreground confusion noise for LISA [20,27] and Taiji [28]. For the space-based GW mission, the confusion noise has three main components: short-period galactic binaries that are mostly from the white dwarf binaries (WDBs), short-period extragalactic binaries and compact objects (white dwarf, neutron star, stellar black hole) captured by MBHs [20,43]. Among these components, the largest one is the galactic WDB background generated by millions of WDBs in the milky way. As shown in Fig. 1 of the LISA white paper [20], in the frequency range 3 × 10^−4^–3 × 10^−3^ Hz, the galactic WDB background confusion noise could exceed the LISA instrumental noise. It is about^1^ 2 × 10^−20^–6 × 10^−19^ Hz^−1/2^. However, from Fig. 5, one can see that the confusion noise level is about 3–4 orders of magnitude smaller than the targeted signals (the thick coloured curves are about a few 10^−16^ Hz^−1/2^). Hence, we argue that it is safe to neglect this component in the PSD.

### Galaxy localization

After generating GW signals, we need to firstly determine the CBC spatial localization volumes based on the GW measurement uncertainties. The simulated MBHB mergers are placed in the three-dimensional space spanned by the GW luminosity distance and sky direction angles, (log *d*_*L*_, α, δ). By marginalizing over other six model parameters, we get the three-dimensional covariance matrix, **Cov**[log *d*_*L*_, α, δ], of the source location parameters. The probability density function of the source localization can be written as

(13) \begin{eqnarray*} f(\log (d_L), \alpha , \delta )&=& C \exp \bigg \lbrace -\frac{1}{2}\Delta \theta ^{T}{\bf Cov}\nonumber\\ &\times &[\log (d_L), \alpha , \delta ]\Delta \theta \bigg \rbrace .\nonumber\\ \end{eqnarray*}

Diagonalize the three-dimensional localization covariance matrix [44,45] to obtain

(14) \begin{eqnarray*} {\bf Cov}^{\prime }(x,y,z)&=&({\bf v_1,v_2,v_3})^{T} {\bf Cov}[\log (d_L), \alpha , \delta ]\nonumber\\ &&\times\,\,({\bf v_1,v_2,v_3})\nonumber \\ &=& \left(\begin{array}{ccc}\lambda _1&\quad 0&\quad 0\\ 0&\quad \lambda _2 &\quad 0\\ 0&\quad 0 &\quad \lambda _3 \end{array}\right), \end{eqnarray*}

where (λ_1_, λ_2_, λ_3_) and (**v_1_, v_2_, v_3_**) are the eigenvalues and eigenvectors of the original covariance **Cov**[log (*d*_*L*_), α, δ]. The orthogonal coordinates (*x*, *y*, $z$) are linearly related with the original coordinates via the rotation

(15) \begin{equation*} \left(\begin{array}{c}x\\ y\\ z \end{array}\right) =({\bf v_1,v_2,v_3})^{T} \left(\begin{array}{c}\log (d_L)\\ \alpha \\ \delta \end{array}\right). \end{equation*}

With the orthogonal coordinates, the probability density function of the source location can be simplified as

(16) \begin{eqnarray*} f(x,y,z)&=& C\exp \bigg \lbrace -\frac{1}{2}\bigg [\frac{(x-\mu _x)^2}{\lambda _1}\nonumber\\ &+& \frac{(y\!-\!\mu _y)^2}{\lambda _2} \!+\!\frac{(z\!-\!\mu _z)^2}{\lambda _3}\bigg ]\bigg \rbrace ,\nonumber\\ \end{eqnarray*}

where (μ_*x*_, μ_*y*_, μ_$z$_) represent the coordinates of the simulated MBHBs and *C* is a normalization factor. This is a chi-square distribution with 3 degrees of freedom. Then we can draw an ellipsoid in (*x*, *y*, $z$) space as

(17) \begin{equation*} \frac{(x-\mu _x)^2}{\lambda _1}+\frac{(y-\mu _y)^2}{\lambda _2}+\frac{(z-\mu _z)^2}{\lambda _3}=\chi ^2 \end{equation*}

with a given confidence level that is characterized by the value of χ^2^. The volume enclosed by the ellipsoids is proportional to the CBC localization probability. In this work, we draw the ellipsoid with a $99\%$ confidence level, which corresponds to χ^2^ = 11.34 according to three-dimensional chi-square statistics.

**Figure 6.**
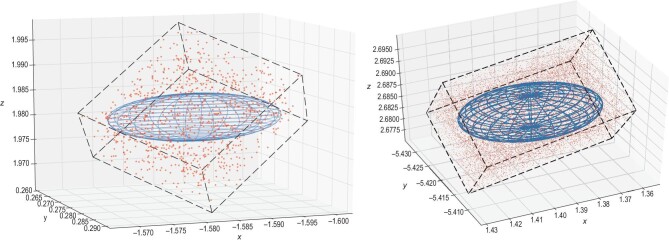
Examples of CBC spatial localizations in the LISA-Taiji network. The blue nested ellipsoids are the $99\%$ confidence regime for CBC localization. The red points are the galaxy samplings. The left and right ellipsoids enclose 185 and 3505 galaxies, respectively.

Then we populate the host galaxy candidates around the targeted ellipsoids. To make sure that the galaxy samplings can cover the targeted ellipsoids, we sample the galaxy in the 4σ ($99.99\%$) confidence regimes. The galaxies are uniformly sampled in the co-moving volume with a number density of 0.02 Mpc^−3^, according to the model [24]. In Fig. 6, we show two examples of CBC spatial localizations in the LISA-Taiji network. The left and right ellipsoids enclose 185 and 3505 galaxies, respectively. The background gray axes are the orthogonal coordinates (*x*, *y*, $z$). The foreground black frames are the original (log *d*_*L*_, α, δ) coordinates. The blue nested ellipsoids are the $99\%$ confidence regimes for CBC localization. The red points are the galaxy samplings. We assume that all galaxy redshifts can be measured with negligible errors. This is a reasonable assumption compared with the luminosity distance errors obtained by a GW measurement. The reasons are as follows. For diamond events, due to the perfect sky localization, we are able to conduct the spectroscopic follow-up. In this case, we safely neglect the redshift uncertainty. For the other types of event, once we consider the clustering effect, it will help the determination of the redshift. Instead of finding the correct host galaxy, we can search for the brightest central galaxy in the clusters where the true host resides. In this case, we can conduct a photometric observation to the larger volume. As predicted for the Vera Rubin Observatory, previously referred to as the Large Synoptic Survey Telescope [46,47], in the redshift range 0 < $z$ < 4 the photometric redshift errors, σ_$z$_/(1 + $z$), must be smaller than 0.05, with a goal of 0.02. The corresponding number for WFIRST (now renamed as the Roman Space Telescope) [48] is about 0.002.

### Hubble parameter estimation

Finally, we estimate the posterior probability distribution of *H*_0_ given both GW data (*d*_GW_) and EM counterpart data (*d*_EM_). According to Bayes’ theorem, the posterior of a single CBC event is

(18) \begin{equation*} p(H_0|d_{\rm GW},d_{\rm EM})=\frac{p(d_{\rm GW},d_{\rm EM}|H_0)p(H_0)}{\beta (H_0)}, \end{equation*}

where *p*(*H*_0_) represents for the prior probability of *H*_0_ and β(*H*_0_) for the evidence. Since the two measurements are independent, we treat the joint GW and EM likelihood, *p*(*d*_GW_, *d*_EM_|*H*_0_), as the product of two individual likelihoods [15,49]. We marginalize over all other variables except for the luminosity distance *d*_*L*_, the solid angle $\hat{\Omega }_{\rm GW}$ of the GW source, the true host galaxy redshift $z$_*i*_ and its solid angle Ω_*i*_. Finally, the joint likelihood for *H*_0_ can be written as

(19) \begin{eqnarray*} &&\!\!\!\! p(d_{\rm GW},d_{\rm EM}|H_0)\nonumber\\ &&\propto\sum _i w_i\iint\!\!\iint p(d_{\rm GW}|d_L,\hat{\Omega }_{\rm GW})\nonumber\\ &&\times\,\,p(d_{\rm EM}|z_i,\Omega _i)\delta (d_L-d_L(z_i,H_0)) \nonumber \\ &&\times\,\, \delta (\hat{\Omega }_{\rm GW}-\Omega _i)p_0(z_i,\Omega _i)dd_Ld\hat{\Omega }_{\rm GW}dz_id\Omega _i,\nonumber\\ \end{eqnarray*}

where the $w$_*i*_ are the weights of each galaxy. Since we do not use other galaxy properties besides their redshifts, we set the weighting factor equal to unity for all galaxies. As mentioned before, we assume that galaxies are uniformly distributed in the co-moving volume. Hence, the prior, *p*($z$_*i*_, Ω_*i*_), for the galaxy redshift space distribution can be written as [49]

(20) \begin{equation*} p_0(z_i,\Omega _i)\propto \frac{1}{V_{\rm max}} \frac{d^2V}{dz_id\Omega } \propto \frac{1}{V_{\rm max}} \frac{\chi ^2(z_i)}{H(z)}, \end{equation*}

where χ($z$) is the co-moving distance to the galaxy.

Assuming that we precisely know the galaxy redshift space position ($z$_*i*_, Ω_*i*_), we can express the EM counterpart likelihood as the product of delta functions:

(21) \begin{equation*} p(d_{\rm EM}|z_i,\Omega _i)\!\propto\! \prod \delta (z_{i,{\rm obs}}-z_i) \delta (\Omega _{i,{\rm obs}}-\Omega _i). \end{equation*}

The GW likelihood, $p(d_{\rm GW}|d_L,\hat{\Omega }_{\rm GW})$, can be calculated according to Equation (13). Therefore, the final posterior for *H*_0_ becomes [44]:

(22) \begin{eqnarray*} p(H_0|d_{\rm GW},d_{\rm EM})&\propto &\frac{p(H_0)}{\beta (H_0)} \sum p(d_{\rm GW}|d_L\nonumber\\ &&\times (z_i,H_0),\Omega _i)p_0(z_i,\Omega _i). \nonumber\\ \end{eqnarray*}

## Supplementary Material

nwab054_Supplemental_File

## Footnotes

1 The vertical axis in Fig. 1 of the LISA white paper [20] is different from that in Fig. 5. One has to divide the former by a factor $\sqrt{f}$ to convert it into the latter.
