# Supplementary material for: Hubble parameter estimation via dark sirens with the LISA-Taiji network
Source: Natl Sci Rev. 2021 Apr 1;9(2):nwab054. doi: 10.1093/nsr/nwab054 (PMC8863402; doi:10.1093/nsr/nwab054)
Supplement: nwab054_Supplemental_File [file nwab054_supplemental_file.pdf]

Supplementary Data

Renjie Wang[1], Wen-Hong Ruan[2,3], Qing Yang[4], Zong-Kuan Guo[2,3,5], Rong-Gen Cai[2,3,5], Bin Hu[1,*]

[1]Department of Astronomy, Beijing Normal University, Beijing, 100875, China
[2]CAS Key Laboratory of Theoretical Physics, Institute of Theoretical Physics, Chinese Academy of Sciences, P.O. Box 2735, Beijing 100190, China
[3]School of Physical Sciences, University of Chinese Academy of Sciences, No. 19A Yuquan Road, Beijing 100049, China
[4]College of Engineering Physics, Shenzhen Technology University, Shenzhen, 518118, China
[5]School of Fundamental Physics and Mathematical Sciences, Hangzhou Institute for Advanced Study, University of Chinese Academy of Sciences, Hangzhou 310024, China

E-mail: bhu@bnu.edu.cn

Contents

1 SUPPLEMENTARY DATA 1

1 SUPPLEMENTARY DATA

In the supplements, we provide the detailed statistics for one typical 5-year simulations. Figure S1 shows the H_0 estimations from all the qualified dark sirens for a 5-year observation. The left and right panels are for Taiji-only and the LISA-Taiji network, respectively. Figure S2 shows the H_0 estimation results from the joint green and blue events. Table S1 summarizes the detailed statistics in the Taiji-only 5-year mission. Table S2 summarizes the detailed statistics in the LISA-Taiji 5-year mission. Table S3 summarizes the averaged event numbers over 40 sets of simulations, for each of the categories (diamond, gold, green, blue), mission configurations (Taiji-only, LISA-Taiji), MBH formation models (PopIII, Q3d, Q3nod) and observation time (1-year, 3-year, 5-year).

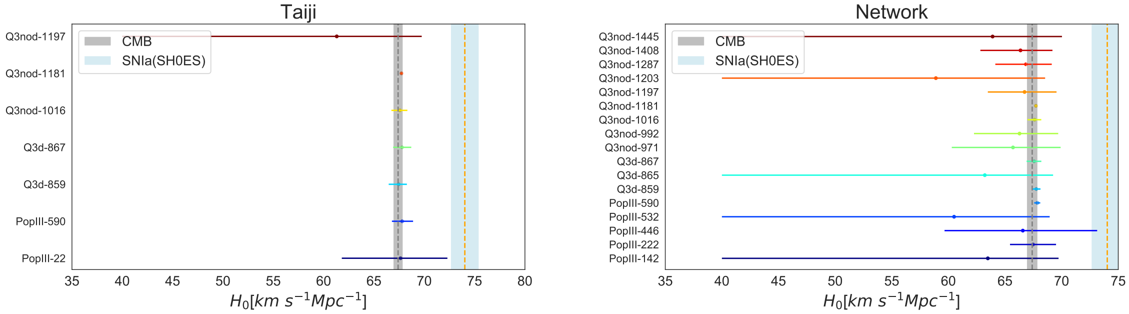

Figure S1: **H_0 estimations from all the qualified dark sirens after 5-year observation.** The left and right panels are for Taiji-only and the LISA-Taiji network, respectively. The vertical grey ($H_0 = 67.4 \pm 0.5$ km s^{-1} Mpc^{-1}) and cyan ($H_0 = 74.03 \pm 1.42$ km s^{-1} Mpc^{-1}) bands denote the present H_0 results from cosmic microwave background (Planck) and SNIa (SH0ES), respectively.

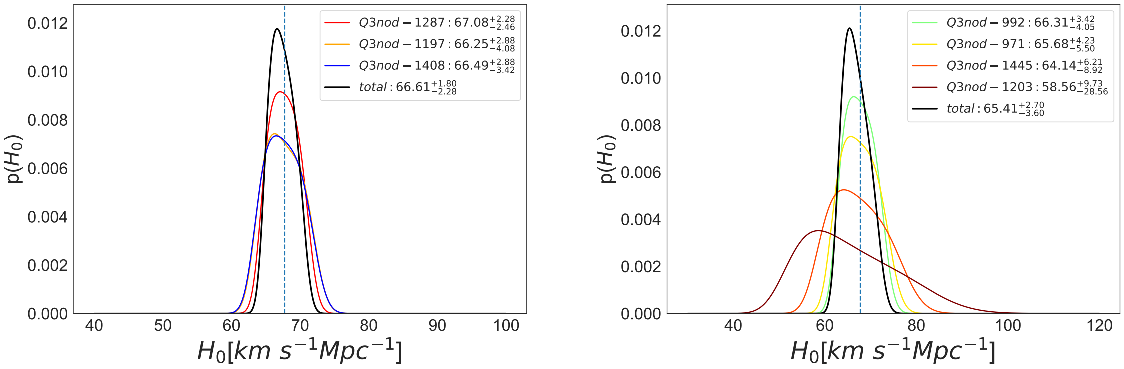

Figure S2: **Joint H_0 results from all the qualified green and blue events.**

Table S1: Taiji dark sirens events

event ID	PopIII-222	PopIII-590	Q3d-859	Q3d-867	Q3nod-1016	Q3nod-1181	Q3nod-1197
H_0	$67.64^{+4.66}_{-5.83}$	$67.81^{+1.08}_{-1.02}$	$67.45^{+0.83}_{-0.98}$	$67.81^{+0.90}_{-0.90}$	$67.58^{+0.75}_{-0.84}$	$67.74^{+0.10}_{-0.10}$	$61.31^{+8.44}_{-21.31}$
rank	blue	green	green	green	green	diamond	blue
$\sigma_{H_0}/H_0[\%]$	6.87	1.60	1.22	1.33	1.10	0.15	12.46
# possible host	6	1022	1818	13646	2376	1	101824
$M_c[M_\odot]$	2810.2	102624.6	544403.5	1786064.9	3136410.5	22196.1	108457.2
redshift	0.13	0.31	0.35	0.39	0.31	0.01	1.19
d_L[Gpc]	$0.6311^{+0.0130}_{-0.0130}$	$1.6676^{+0.0259}_{-0.0259}$	$1.9202^{+0.0185}_{-0.0185}$	$2.1801^{+0.0334}_{-0.0334}$	$1.6676^{+0.0101}_{-0.0101}$	$0.0446^{+3.916\times10^{-5}}_{-3.916\times10^{-5}}$	$8.4735^{+0.3166}_{-0.3166}$
inclination [rad]	0.529608	1.678562	1.560729	1.734877	1.792044	0.239651	1.375129
sky area [$\deg^2$]	0.005410083	0.515173338	0.916650806	4.761781352	1.795273896	4.85×10^{-5}	0.298690746
SNR w/ lensing	7.6289835×10^2	5.6381308×10^2	4.1690040×10^2	4.2679994×10^2	6.2915549×10^2	1.1296812×10^5	1.1002939×10^2
SNR w/o lensing	7.8537225×10^2	1.4506480×10^3	2.5970506×10^3	1.6703106×10^3	3.0178431×10^3	1.2227127×10^5	3.1135649×10^3

Table S2: LISA-Taiji network dark sirens events

event ID	PopIII-142	PopIII-222	PopIII-446	PopIII-532	PopIII-590	Q3d-859
H_0	$63.48^{+6.25}_{-23.48}$	$67.47^{+2.03}_{-2.03}$	$66.57^{+6.57}_{-6.91}$	$60.50^{+8.44}_{-20.50}$	$67.85^{+0.26}_{-0.28}$	$67.77^{+0.36}_{-0.37}$
rank	blue	green	blue	blue	diamond	gold
$\sigma_{H_0}/H_0[\%]$	9.22	3.00	9.69	12.46	0.38	0.54
# possible host	35881	1	2	94844	3	20
$M_c[M_\odot]$	59268.6	2810.2	6164.1	88554.9	102624.6	544403.5
redshift	1.72	0.13	0.14	2.24	0.31	0.35
d_L[Gpc]	$13.3539^{+0.2900}_{-0.2900}$	$0.6311^{+0.0079}_{-0.0079}$	$0.6840^{+0.0280}_{-0.0280}$	$18.4550^{+0.5530}_{-0.5530}$	$1.6676^{+0.0023}_{-0.0023}$	$1.9202^{+0.0028}_{-0.0028}$
inclination [rad]	2.086382	0.529608	0.3583	1.076666	1.678562	1.560729
sky area [deg^2]	0.087674	0.000192	0.000623	0.114884	0.004459	0.016898
SNR w/ lensing	1.2090829×10^2	1.1229768×10^3	8.9548935×10^2	8.8478991×10^1	8.9709385×10^2	6.9977139×10^2
SNR w/o lensing	3.0284694×10^2	1.1672291×10^3	9.4983829×10^2	2.5121068×10^2	2.7006799×10^3	4.8139297×10^3

event ID	Q3d-865	Q3d-867	Q3nod-971	Q3nod-992	Q3nod-1016	Q3nod-1181
H_0	$63.22^{+6.03}_{-23.22}$	$67.57^{+0.64}_{-0.66}$	$65.71^{+4.20}_{-5.41}$	$66.28^{+3.42}_{-4.02}$	$67.61^{+0.59}_{-0.62}$	$67.73^{+0.08}_{-0.08}$
rank	blue	gold	blue	blue	gold	diamond
$\sigma_{H_0}/H_0[\%]$	8.90	0.95	6.21	5.04	0.87	0.11
# possible host	89504	185	39027	54407	202	1
$M_c[M_\odot]$	192432.6	1786064.9	147880.5	635582.8	3136410.5	22196.1
redshift	2.03	0.39	1.03	1.01	0.31	0.01
d_L[Gpc]	$16.3646^{+0.3750}_{-0.3750}$	$0.0824^{+0.0060}_{-0.0060}$	$7.0872^{+0.1421}_{-0.1421}$	$6.9174^{+0.0975}_{-0.0975}$	$1.6676^{+0.0039}_{-0.0039}$	$0.0446^{+2.1963\times10^{-5}}_{-2.1963\times10^{-5}}$
inclination [rad]	1.232853	1.734877	1.046315	1.146302	1.792044	0.239651
sky area [deg^2]	0.175161	0.082398	0.337444	0.600698	0.185151	2.55×10^{-7}
SNR w/ lensing	9.4601071×10^1	6.0290934×10^2	2.1465086×10^2	2.1095443×10^2	8.3236940×10^2	1.4789984×10^5
SNR w/o lensing	3.9412333×10^2	3.5984501×10^3	2.0724458×10^3	2.7899098×10^3	4.5630742×10^3	1.5900531×10^5

event ID	Q3d-1197	Q3nod-1203	Q3nod-1287	Q3nod-1408	Q3nod-1445
H_0	$66.73^{+2.82}_{-3.24}$	$58.89^{+9.65}_{-18.89}$	$66.85^{+2.28}_{-2.70}$	$66.37^{+2.82}_{-3.54}$	$63.90^{+6.13}_{-23.90}$
rank	green	blue	green	green	blue
$\sigma_{H_0}/H_0[\%]$	4.17	14.24	3.37	4.17	9.04
# possible host	3505	242291	20541	20750	30165
$M_c[M_\odot]$	108457.2	46212.4	387382.6	38624.7	58704.5
redshift	1.19	2.18	1.1	1.63	1.37
d_L[Gpc]	$8.4735^{+0.0772}_{-0.0772}$	$17.8539^{+0.7211}_{-0.7211}$	$7.6877^{+0.0920}_{-0.0920}$	$12.4990^{+0.2487}_{-0.2487}$	$10.0862^{+0.1965}_{-0.1965}$
inclination [rad]	1.375129	0.924557	1.811709	1.55503	0.955186
sky area [deg^2]	0.035014	0.257477	0.292858	0.12561	0.105388
SNR w/ lensing	1.6205164×10^2	9.8563823×10^1	1.9153599×10^2	1.1504490×10^2	1.7300351×10^2
SNR w/o lensing	4.0766870×10^2	2.9280596×10^2	1.6681709×10^3	2.0130234×10^2	6.5965008×10^2

Table S3: **The averaged classified dark sirens event number over** 40 **sets of simulations.** The numbers in the parentheses of the last columns are the total CBC events.

model	detector	observation time	$\sigma_{H_0} < 0.5\%$	$0.5\% < \sigma_{H_0} < 1\%$	$1\% < \sigma_{H_0} < 5\%$	$\sigma_{H_0} > 5\%$
PopIII	network	1yr	0.125	0.05	0.01	0.45 (166.725)
		3yrs	0.375	0.05	1.275	2.775 (499.3)
		5yrs	0.375	0.2	0.875	3.6 (833.55)
	taiji	1yr	0.05	0.0	0.05	0.0 (166.9)
		3yrs	0.175	0.15	0.175	0.325 (500.5)
		5yrs	0.2	0.125	0.225	0.225 (834.45)
Q3d	network	1yr	0.05	0.0	0.1	0.175 (7.85)
		3yrs	0.05	0.075	0.35	0.7 (23.525)
		5yrs	0.125	0.125	0.375	1.225 (39.375)
	taiji	1yr	0.05	0.0	0.025	0.1 (7.925)
		3yrs	0.025	0.025	0.15	0.075 (23.8)
		5yrs	0.05	0.05	0.25	0.2 (39.65)
Q3nod	network	1yr	0.05	0.0	0.45	0.7 (115.5)
		3yrs	0.4	0.2	7.2	15.6 (344.2)
		5yrs	0.65	0.25	4.05	8 (576.05)
	taiji	1yr	0.05	0.0	0.05	0.15 (115.9)
		3yrs	0.275	0.075	0.525	1.4 (348.125)
		5yrs	0.325	0.25	0.575	1.725 (579.85)
